# Hemodialysis Session Duration, Ultrafiltration Rate, Interdialytic Weight Gain and their Association with Albumin in Patients on Intermittent Hemodialysis in a Regional Dialysis Unit: A Cross-sectional Study

**DOI:** 10.7759/cureus.3794

**Published:** 2018-12-28

**Authors:** Adeel Rafi Ahmed, Ala Eldin Abdalla, Muniza Satti, Junaid Qazi, David Lappin

**Affiliations:** 1 Nephrology, Galway University Hospital, Galway, IRL; 2 Internal Medicine, National University of Ireland, Galway, IRL

**Keywords:** hemodialysis session duration, albumin, end stage renal disease, mortality, ultra-filtration, interdialytic weight gain.

## Abstract

Aim

Several studies suggest that a low pre-dialysis serum albumin level (<40g/L) is associated with increased mortality in dialysis patients. The objective of this study was to assess if hemodialysis session duration (HSD), ultrafiltration rate (UFR) and interdialytic weight gain percentage (IDWG%) are associated with pre-dialysis serum albumin levels (markers of all-cause mortality), thus influencing mortality.

Method

This is a cross-sectional analytical study in which data were collected from a regional cohort of 59 prevalent chronic hemodialysis patients using a national electronic database (eMED). Continuous data were analyzed using a regression model to assess for an association between HSD, IDWG% and UFR with albumin levels.

Results

Fifty-six patients were included in the study. Multiple linear regression models demonstrated a cross-sectional association between longer HSD and higher serum albumin levels and a statistically significant positive correlation (*r *= 0.353; *p *< 0.05). No significant association of UFR (*p *= 0.169) and IDWG% (*p *= 0.549) with albumin was observed. Mean albumin was 38.07 ± 3.96 g/L in the HSD <240 min group compared to 40.50 ± 2.83g/L in the HSD ≥240 min group which was statistically significant (*p *< 0.05).

Conclusion

Longer HSD has a cross-sectional association with higher pre-dialysis serum albumin with patients having HSD ≥240 min demonstrating the highest levels of serum albumin. Our study suggests longer HSD may improve mortality in the dialysis population.

## Introduction

Hemodialysis (HD) adequacy is primarily measured using small solute clearance based on the urea kinetic modelling (UKM) [1]. However, HD adequacy is a multifaceted process and UKM in isolation can be misleading when assessing HD patients. The landmark Hemodialysis Study (HEMO) did not demonstrate any significant difference in mortality or cardiac events between mean single pooled Kt/V (spKt/v) 1.3 (low dose) vs 1.7 (high dose) with a mean hemodialysis session duration (HSD) of 190 ± 23 vs 219 ± 23 min, respectively [2]. Despite improvements in dialysis technology, the mortality rates remain high in the dialysis population compared to the age-matched general population [3]. Large observational studies have identified short HSD, high ultrafiltration rates (UFR) and high intradialytic weight gain (IDWG) as important independent markers of mortality and adverse cardiovascular outcomes [4-7].

In particular, albumin <40g/L is a strong predictor of all-cause mortality in HD patients, representing poor nutrition and inflammation [7-11]. We hypothesize long HSD, slow UFR and lower IDWG% are associated with higher serum albumin levels, thus indicating better efficacy of dialysis and providing mortality benefits. 

## Materials and methods

We conducted a cross-sectional analytical study to investigate the association between HSD, UFR and IDWG% with pre-dialysis serum albumin levels (a marker of all-cause mortality) in patients at a regional dialysis unit in County Galway, Republic of Ireland. 

We identified all dialysis patients who had thrice weekly HD, aged ≥ 18 years, had a single pooled Kt/V ≥1.2 and ≥3 months on dialysis. Our primary response variable was serum albumin as a surrogate endpoint for dialysis adequacy and all-cause mortality.

Data were obtained retrospectively from a regional cohort of prevalent chronic HD patients using a national electronic database (eMED). For each patient, data were collected through a standardized printed form that was subsequently computerized.

Summary statistics of continuous data were given as mean values, standard deviations (SD) and proportions (percentage) for categorical data. We used a stepwise multiple linear regression model to determine the association of each variable with albumin and to adjust for potential confounding variables. A *p*-value of <0.05 was considered as statistically significant. Statistical analysis was carried out using Minitab® version 17.0.

## Results

Fifty-six patients were included in the study with a mean age of 66 ± 16 years and a median dialysis vintage of 24 months. Three patients were excluded due to the missing data required to complete statistical analysis.

Patient characteristics

The gender representation in the dialysis cohort was 33 (59%) males and 23 (41%) females. Majority of the participants had a double-lumen cuffed catheter as a mode of dialysis access (*n *= 39). The mean HSD was of 220 ± 20.39 min. Approximately 46.43% patients achieved HSD ≥240 min with a mean of 241 ± 3.73 min. The dialysis population demonstrated mean values of UFR 7.91 ml/hr/kg±2.73, IDWG% 2.74 ± 1.44, Kt/V 1.33 ± 0.14 and albumin 39 g/L ±3.66. The mean pre-HD phosphate and systolic blood pressures were 1.45 ± 0.40 mmol/L and 136.98 ± 26.10 mmHg, respectively with no significant difference between HSD ≥240 min and <240 min groups.

Table 1 shows the baseline characteristics of the dialysis population. 

**Table 1:** Baseline characteristics ^1^patient variables given as mean ± standard deviation (SD) where applicable; ^2 ^Median DM: diabetes mellitus, HSD: hemodialysis session duration, IDWG: interdialytic weight gain; UF: ultrafiltration, UFR: ultrafiltration rate, HD: hemodialysis, SBP: systolic blood pressure, AVF: arteriovenous fistula, K: dialyzer clearance of urea, T: dialysis time, V: volume of urea distribution

Variables^1^	HSD <240 min (*n *= 30)	HSD ≥240min (*n *= 26)	Significance of difference (*p*-value)	Total
Age (years)	68 ± 16	63 ± 16		66 ± 16
Male	11	22		33
Female	19	4		23
DM	8	12		20
HSD (min)	202 ± 9	241 ± 3.73	<0.05	220 ± 20.39
IDWG (Kg)	1.67 ± 0.98	2.27 ± 0.98	<0.05	1.95 ± 1
IDWG (%)	2.65 ± 1.55	2.83 ± 1.42	0.65	2.74 ± 1.44
UF (L)	1.85 ± 0.64	2.45 ± 0.65	<0.05	2.13 ± 0.71
UFR (ml/h/kg)	8.39 ± 3.09	7.35 ± 2.36	0.16	7.91 ± 2.98
Albumin (g/L)	38.07 ± 3.96	40.50 ± 2.83	<0.05	39.20 ± 3.66
Dialysis vintage (months)^2^	24	12		24
Pre-HD phosphate (mmol/l)	1.47 ± 0.50	1.42 ± 0.28	0.64	1.45 ± 0.40
Pre-HD SBP (mmHg)	134.77 ± 24.84	139.50 ± 27.75	0.51	136.98 ± 26.10
Post-HD SBP (mmHg)	126.63 ± 20.68	131 ± 16.93	0.39	128.66 ± 19
Permacath	21	18		39
AVF	9	8		17
KT/V	1.37 ± 0.15	1.30 ± 0.24	0.206	1.33 ± 0.14

Clinical outcome

There was a significant, positive correlation between increasing HSD and higher serum albumin levels (*r *= 0.353; *p *< 0.05). There was no significant association between UFR (*p *= 0.169) and IDWG% (*p *= 0.549) and albumin levels. Stepwise multiple linear regression was performed to examine the simultaneous effect of six independent continuous variables on serum albumin levels. Included variables were age, HSD, UFR, IDWG%, dialysis vintage and Kt/V. Stepwise selection found HSD was a statistically significant determinant of higher serum albumin levels. Mean albumin was 38.07 ± 3.96 g/L in HSD <240 min group compared to 40.50 ± 2.83g/L in HSD ≥240 min group which was statistically significant (*p *< 0.05).

Table 2 shows the results of the correlations and multiple linear regression model.

**Table 2:** Correlations and multiple linear regression model r: correlation coefficient, S: standard error of the regression, b: standardized regression coefficient, HSD: hemodialysis session duration, IDWG: interdialytic weight gain, UF: ultrafiltration, UFR: ultrafiltration rate, K: dialyzer clearance of urea, T: dialysis time, V: volume of urea distribution

	Correlation				Multivariable Linear Regression (R^2 ^= 23.77%)	
Variable	*r*	*P* value	R^2^ (%)	S	b	*P* value
Age (years)	-0.258	0.055	6.64	15.50	-0.947	0.081
HSD (min)	0.353	0.008	12.49	19.247	0.988	0.047
IDWG (Kg)	0.179	0.187	3.20	1.00958		
IDWG%	0.082	0.549	0.67	1.48		
UF (L)	0.069	0.614	0.47	0.72		
UFR( ml/h/kg)	-0.186	0.169	3.48	2.77	-0.889	0.086
Dialysis Vinatage (months)	0.086	0.529	0.74	22.34	0.649	0.173
KT/V	0.043	0.754	0.18	0.136		

## Discussion

The results demonstrate an association with increasing HSD and higher pre-dialysis serum albumin levels. Subgroup analysis found HSD ≥240 min group had a significantly higher mean serum albumin levels compared to HSD <240 min. These findings suggest dialysis adequacy achieved via longer HSD may provide mortality benefits when using serum albumin as a surrogate marker. No significant association of UFR and IDWG% with albumin was observed in this cross-sectional analysis.

In large observational studies, strongest association with reduced mortality was seen in groups having serum albumin ≥ 40g/L [7,11]. Lower serum albumin levels have also been associated with peripheral vascular disease, chronic obstructive pulmonary disease, cancer and patients using temporary catheters [12]. Serum albumin level <40 g/L is a strong predictor of all-cause mortality in HD patients, representing poor nutrition and inflammation [7-11].

HSD has an important role in dialysis patient survival, which is highlighted by the Tassin experience in France. The dialysis unit in Tassin utilized HSD of ≥480 min, three times per week, in a majority of its patients; approximately 95% of the patient cohort achieved normal blood pressure without antihypertensive medication and had significantly lower mortality and morbidity compared to the data from the United States renal data system [13]. Large observational studies have consistently demonstrated a reduction in all-cause mortality with longer HSD (≥240 min) [4,14-16]. Data from the Dialysis Outcomes and Practice Patterns Study (DOPPS) showed a reduction of 7% in relative risk of mortality for every 30-min additional HSD with lower pre- and post-dialysis systolic blood pressure, greater intradialytic weight loss, higher hemoglobin (for the same erythropoietin dose), serum albumin, potassium and lower serum phosphorus levels and white blood cell counts [4,15]. Longer HSD ≥240 min demonstrated decreased all-cause mortality when compared to HSD <240 min independent of body weight [16].

Our patient data indicated 46.43% patients achieved HSD ≥240 min with an overall mean of 220 ± 20.39 min. A significant correlation (*r *= 0.353; *p *< 0.05) between increasing HSD and higher pre-dialysis serum albumin was observed. This effect could be due to the efficient clearance of uremic toxins with longer HSD, thus improving the inflammatory state of the HD patients. The more effective removal of uremic toxins may also improve appetite, leading to an improved nutritional state demonstrated by higher albumin levels. 

Observational data from landmark studies, HEMO and DOPPS, found a significant association of UFR >10 ml/h/kg and all-cause mortality [15,17]. One observational study demonstrated higher all-cause mortality at UFR >12.37 ml/h/kg [18]. The largest observational study to date involving 118,294 chronic HD patients adjusted to body weight, body mass index (BMI) and body surface area (BSA) showed a UFR >10 ml/h/kg was associated with increased all-cause mortality [5].

A UFR >10 ml/h/kg was prevalent in only 16.90% of our patient cohort with an overall mean of 7.88 ml/h/kg ±2.73. This may be the reason that no independent correlation with serum albumin levels could be established in our relatively small patient cohort. It is also plausible that UFR has no association with albumin, and the increased mortality observed with high UFR is primarily due to the physiological stress on the myocardium. 

Data regarding IDWG% has been associated with better nutrition but increased mortality in dialysis patients, thus leading to a divided opinion on its management [19-23]. Recent, larger and more robust studies have demonstrated a steady association with high IDWG% (or IDWG > 3 kg) ≥4 and increased mortality and hospitalization, independent of UFR and HSD [6,19,20,24]. One explanation to reconcile these relatively opposing observations is the cohort study consisting of more than 50,000 HD patients from 25 countries which showed a steady decline in IDWG% most significant at approximately 20 weeks prior to death; however, patients who survived (more than four years on dialysis) had a relatively stable mean IDWG% between 2.5% and 3.5% (European population) [25].

IDWG% ≥4 was observed in 13.60% of the patients in our cohort with a mean of 2.74 ± 1.44%. There is reasonable observational data to suggest high IDWG% ≥4 is harmful to patients leading to larger volume shifts during dialysis [6,19,20,24]. Small sample size and the majority of the patients achieving IDWG% <4 could have resulted in no significant association being established with serum albumin.

This study has several limitations that need to be considered when interpreting the present results. The resulting sample size is relatively small, and thus, any correlation that may exist in a larger cohort could not be demonstrated. The data collected for the study were from a hospital-based electronic system and all measurements have been taken previously; therefore, we have no control over the data measurement quality, accuracy and the completeness of data. To minimize any error in the data extraction, we used a standardized form. Data from our cohort did not include other inflammatory markers like C-reactive protein, and thus we could not determine its influence on the outcome observed. As with many observational studies, we cannot exclude the possibility of residual confounding due to unknown or unmeasured measured factors. To our knowledge, this is the first study to report the potential beneficial effects of longer HSD on a dialysis population in the Republic of Ireland. This study further asserts the notion of mortality benefits with a longer dialysis time.

## Conclusions

In conclusion, longer HSD has a cross-sectional association with higher pre-dialysis serum albumin levels. Patients having HSD ≥240 min demonstrated the highest levels of serum albumin. Our study suggests longer HSD may improve mortality in the dialysis population. There are many factors that influence shorter HSD, such as patient preference, tolerance and a shortage of nursing staff. Patients need to be counseled regarding the advantages of longer HSD. HSD ≥240 min is achievable in most dialysis units in the Republic of Ireland and certainly, the trend needs to be towards it.
